# Experimental identification of the behaviour of and lateral forces from freely-walking pedestrians on laterally oscillating structures in a virtual reality environment

**DOI:** 10.1016/j.engstruct.2015.09.043

**Published:** 2015-12-15

**Authors:** Mateusz Bocian, John H.G. Macdonald, Jeremy F. Burn, David Redmill

**Affiliations:** a Department of Civil Engineering, University of Bristol, Queen’s Building, University Walk, Bristol BS8 1TR, UK; b Department of Mechanical Engineering, University of Bristol, Queen’s Building, University Walk, Bristol BS8 1TR, UK; c Vibration Engineering Section, College of Engineering, Mathematics and Physical Sciences, University of Exeter, Kay Building, North Park Road, Exeter EX4 4QF, UK; d School of Experimental Psychology, University of Bristol, 12a Priory Road, Bristol BS8 1TU, UK

**Keywords:** CoM, centre of mass, CoP, centre of pressure, FFT, fast Fourier transform, GRF, ground reaction force, HMD, head-mounted display, IPM, inverted pendulum model, MCS, motion capture system, MIV, manipulated independent variable, NTLM, no treadmill lateral motion, NVR, no virtual reality, TLM, treadmill lateral motion, VR, virtual reality, Bridges, Human–structure interaction, Biomechanics, Inverted pendulum pedestrian model, Self-excited forces, Virtual reality environment

## Abstract

Modelling pedestrian loading on lively structures such as bridges remains a challenge. This is because pedestrians have the capacity to interact with vibrating structures which can lead to amplification of the structural response. Current design guidelines are often inaccurate and limiting as they do not sufficiently acknowledge this effect. This originates in scarcity of data on pedestrian behaviour on vibrating ground and uncertainty as to the accuracy of results from previous experimental campaigns aiming to quantify pedestrian behaviour in this case. To this end, this paper presents a novel experimental setup developed to evaluate pedestrian actions on laterally oscillating ground in the laboratory environment while avoiding the implications of artificiality and allowing for unconstrained gait. A biologically-inspired approach was adopted in its development, relying on appreciation of operational complexities of biological systems, in particular their adaptability and control requirements. In determination of pedestrian forces to the structure consideration was given to signal processing issues which have been neglected in past studies. The results from tests conducted on the setup are related to results from previous experimental investigations and outputs of the inverted pendulum pedestrian model for walking on laterally oscillating ground, which is capable of generating self-excited forces.

## Highlights

• A novel setup for investigating pedestrian–structure interaction is presented.
• Foot-placement is the main balance control mechanism on laterally vibrating ground.
• All components of pedestrian force are uncovered, including self-excited forces.
• Inverted pendulum pedestrian model qualitatively captures the nature of forces.
• The ground and visual conditions cause significant changes in pedestrian loading.

## 1 Introduction

A number of attempts to determine pedestrian loading on laterally oscillating bridges in which pedestrian forces are inferred through inverse dynamics, i.e. from analysis of the time histories of the behaviour of full scale bridges during pedestrian occupancy, can be classified as *top-down* modelling approaches. This type of identification process is essentially achieved through decomposition of the coupled crowd-structure system into its more basic components and analysis of their contributions to the system dynamic behaviour. The obtained pedestrian forces come from empirical data hence have an advantage of being based on direct evidence. However, loading models constructed in this manner do not generally have the capability of explaining mechanisms causing lateral instability nor can they be generalised to other structures. The reason for this is the inherent bias of *top-down* modelling approaches towards higher organisational rank, i.e. fitting unknown behaviour of the input drivers to the known performance of the whole system. This favouritism can lead to misinterpretation of the system, in this case the oversimplification of pedestrian behaviour, which is often assumed rather than evidential, and as an effect can obstruct rather than facilitate understanding. This problem is the most relevant to a number of modelling approaches in which synchronisation of a group of pedestrians was proposed as the sole mechanism causing structural instability [1].

Alternative approaches to the pedestrian–structure interaction problem utilise *bottom-up* modelling. *Bottom-up* modelling relies on pre-existing knowledge of elementary components of the system and their behaviour in building of the system model. It therefore fundamentally differs from the *top-down* modelling in that it involves synthesis rather than decomposition. In the context of pedestrian–structure interaction on bridges the constituent components for synthesis are the bridge and the pedestrians. With the modal properties of the bridge usually well defined, either through analytical or experimental methods (i.e. finite element modelling and modal testing, respectively), the veracity of the model depends on accurate characterisation of the ground reaction forces occurring at the interface of these two dynamic subsystems. This critical task, in turn, demands good understanding of pedestrian behaviour and requires separate, purpose-oriented investigations.

Today, the prevailing concept in modelling pedestrian lateral loading is that proposed by Arup [2], derived from analysis of measurements from the dynamic behaviour of the London Millennium Footbridge under the action of crowds, thereby representing the *top-down* modelling approach. It relies on an observation that walking pedestrians, within certain conditions, can be treated as a source of negative damping to the structure. In fact many other models, which are most often based on the notion of synchronisation, are at their culmination simply calibrated to match the results obtained by Arup over a decade ago. Therefore their applicability in predicting structural dynamic behaviour, especially for cases when the structural and crowd characteristics are different than those experienced on the London Millennium Footbridge, can be considered questionable. A better solution to the problem is to develop a fundamental pedestrian model, applicable to walking on vibrating ground, while avoiding any preconceptions of pedestrian behaviour.

### 1.1 A fundamental model of pedestrian behaviour while walking on laterally oscillating ground

Based on a framework introduced by Barker [3], a fundamental pedestrian model for walking on laterally oscillating ground was proposed by Macdonald [4]. He conceived an inverted pendulum pedestrian model (IPM) rooted in the field of biomechanics, representing dynamics of walking in the frontal plane (i.e. vertical plane perpendicular to the direction of progression). As in Barker’s model, the self-excited forces were generated based on an assumption of the pedestrian walking frequency being unaffected by the bridge motion. Unlike in Barker’s model, Macdonald’s model included allowance for the effect of the forces from bridge motion on the pedestrian mass hence accounted for the full bi-directional pedestrian–structure interaction. Because step width modulation is known to be the most important balance control strategy in the presence of lateral gait perturbation while walking on stationary ground [5], a stepping control law was adopted in the model based on findings by Hof et al. [6]. According to that control law a pedestrian will respond to lateral perturbation by augmenting the step width proportionally to the lateral velocity of the CoM and a constant offset, at the instance of foot placement.

Fourier decomposition of the pedestrian lateral force in the presence of lateral bridge motion with frequency $f_{b}$ obtained from the IPM revealed that in addition to components of the force characteristic for walking on stationary ground, at stride frequency $f_{p}$ and its odd harmonics, other self-excited (or motion-dependent) force components which lay symmetrically around these harmonics at ${\mathrm{jf}}_{p}\pm \delta f$, where j is an odd integer and $\delta f=|f_{p}-f_{b}|$ is the beating frequency, are present in the frequency spectrum [4]. The component of the self-excited forces at the bridge vibration frequency, $F_{L\text{,}f_{b}}$, is of upmost importance as it can input energy into (or extract energy from) the relevant vibration mode.

The premise of the bridge motion not affecting pedestrian walking frequency, underlying Barker’s [3] and Macdonald’s [4] models, and the observation that unsynchronised pedestrians’ loading is able to lead to structural instability are corroborated by the results of measurements of dynamic behaviour of the Changi Mezzanine Bridge (CMB) at the Singapore’s Airport by Brownjohn et al. [7] and on the Clifton Suspension Bridge by Macdonald [8]. No evidence of synchronisation was observed on these bridges during divergent amplitude vibration periods. In agreement with the observation made by Arup on the London Millennium Footbridge [2], the amplitude of the effective pedestrian lateral force on each mode was found to be proportional to the amplitude of the local lateral velocity of the deck. As with any tests on full-scale structures, only certain parameter ranges could be investigated, determined by the intrinsic dynamic characteristics of the structures.

Further theoretical analyses of the model were conducted by Bocian et al. [9], [10], McRobie [11] and McRobie et al. [12]. A few experimental studies have also been conducted which give some support to the applicability of the IPM proposed by Macdonald [4].

### 1.2 Experimental identification of self-excited forces on laterally oscillating structures

To date, the most valuable effort of measuring lateral pedestrian forces in the presence of lateral bridge motion in a laboratory environment has been made by Ingólfsson et al. [13] who conducted an extensive experimental campaign on a slightly modified setup of Pizzimenti and Ricciardelli [14]. Adopting the framework of Pizzimenti and Ricciardelli [14], the self-excited component of the force at the bridge vibration frequency was quantified in terms of additional damping and mass (rather than stiffness as in [14]) to the structure for combinations of vibration frequency and amplitude in the ranges of 0.33–1.07 Hz and 4.5–48 mm, respectively. Large scatter of the results was observed, particularly at low vibration amplitudes, while $F_{L\text{,}f_{b}}$ was found to depend on the vibration frequency and amplitude. Subsequently, a stochastic model of pedestrian loading was proposed based on the experimental findings [15]. Although significant insight into the interaction between pedestrians and laterally oscillating bridges was gained, many issues remained unresolved, mainly due to the shortcomings of the experimental setup and the experimental protocol, thus questioning the accuracy of the derived loading model and its predicted structural response. Specifically, the following main points (hereafter referred to as Points 1–3) were not addressed:

1. The tests were conducted in a laboratory environment with an abundance of stationary visual reference cues (as the walker on a treadmill is generally stationary relative to the environment). However, normal overground walking causes a specific optic flow, i.e. a pattern of apparent motion of the physical world perceived by an observer through their visual system due to self-motion, with a focus of radial expansion fixed at the point towards which the observer is moving [16]. Additional information to the locomotor system (i.e. organ system allowing movement to be generated using muscular and skeletal systems) is provided by motion parallax, i.e. apparent relative motion of objects away from the observer against the rest of the visual field caused by self-motion of the observer [17]. Both of these mechanisms through which visual perception is realised have been found to contribute to postural sway during walking and to influence stability [18]. As observed in the context of walking by Guerin and Bardy [19], dynamic systems theory dictates that, considering this adaptive nature of human gait, lack of congruence between visual and non-visual information can have an effect in that the locus of attracting and repelling states of the system can be changed. In other words, pedestrian behaviour (hence loading) can depend on the quality of visual information available to the walker. A lack of compatibility between visual and non-visual sensory information can be considered an important procedural shortcoming, especially when studying the stability of gait, which is an underlying driver of pedestrian–structure interaction on lively bridges.
2. It is well known that human (and more generally, animal) locomotion is adaptive in its nature, i.e. walking patterns can be adjusted in response to internal or external constraints, and it is optimised according to circumstantial requirements [20]. Therefore, restrictions imposed on the ability to adjust gait freely can prevent natural behaviour. These implications are likely to arise in tests on perturbed gait from imposing constant speed of the treadmill belt and/or focusing on self-selected speed for walking on stationary ground. This occurred during the tests and might have biased the results.
3. The duration of each test was fixed at 30 s for any combination of lateral vibration frequency and amplitude. As the pedestrian frequency was in most cases unaffected by the lateral treadmill motion, it is unlikely that the relative phases between the motion of the bridge and the pedestrian loading covered the full actual range in a representative manner. Therefore, the calculated magnitudes of forces might not correspond to the average expected values (see Section 3.2.3 for further discussion of this issue).

It is hypothesised here that the issues raised above might have biased the results of past experimental studies. Perceptual and control issues (see Points 1 & 2) might have also hindered results from recent tests by Carroll et al. [20], [21] who essentially rebuilt the experimental setup developed by Pizzimenti and Ricciardelli [14].

### 1.3 Scope of the current study

Motivated by an ambition to validate and potentially improve the results from previous experimental campaigns and further advance the IPM, a new experimental setup was designed and built. A biologically-inspired approach was adopted in its development, relying on appreciation of operational complexity of biological systems, in particular their adaptability and control requirements. This was achieved by allowing the pedestrians walking on an instrumented treadmill to adjust their walking speed freely, by means of motor speed feedback control mechanism, and providing a visual environment representative of walking on a real bridge, by means of an immersive and interactive virtual reality environment. To the best of the authors’ knowledge, this is the first time virtual reality technology has been used in research on the behaviour of people in the built environment in the context of vibration serviceability of structures. It was not the intention of the current study to provide a comprehensive set of data for a wide range of lateral bridge vibration frequencies and amplitudes, which would require very many tests, but rather to focus on the uncertainties associated with perceptual, control and signal processing issues that might have hindered the results of previous laboratory investigations. Therefore, one combination of treadmill vibration frequency and amplitude was chosen for this purpose and a thorough experimental study was conducted regarding the other issues. The significance of the chosen set of vibratory conditions is that the amplitude and frequency fall within the ranges observed on real structures during periods of lateral instability and the frequency is in the centre of the range of typical stride frequencies for normal walking. Considering the above, adaptations in pedestrian walking behaviour invoked by laterally oscillating ground are revealed. The pedestrian lateral force is rigorously analysed allowing all components of pedestrian loading to be identified and compared to the output of the IPM. Finally the effects of visual environment and ground motion on pedestrian gait parameters and self-excited forces are examined and their statistical significance is tested.

## 2 Novel experimental setup

**Fig. 1:**
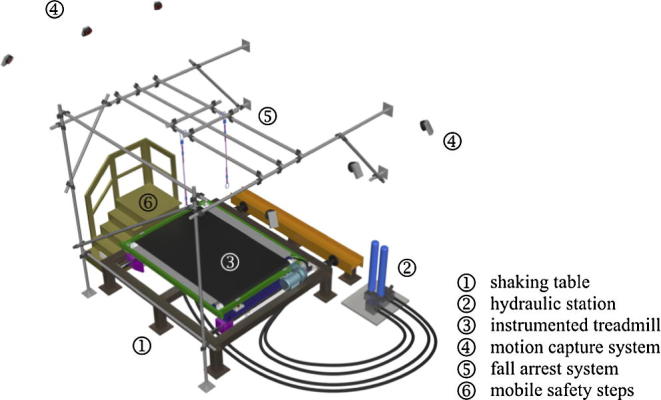
Spatial arrangement of the motion base-mounted instrumented treadmill.

A simple idea underlying the experimental campaign conducted with help of the setup (see Fig. 1) was to evaluate walking on a laterally oscillating structure in laboratory conditions while avoiding the implications of artificiality and allowing for unconstrained gait. An instrumented mechanical treadmill (based on Kistler 9327C piezoelectric force transducers) was chosen for that purpose due to its practical advantages in gait analysis, in particular the ability to measure continuous ground reaction forces (GRFs) without the large space requirement for walkways and without the problems associated with targeting foot placements on force plates. Lateral oscillations were applied by mounting the treadmill on top of a uniaxial hydraulic shaking table. Virtual reality was implemented through a head mounted display (HMD; nVisor SX111) in which an immersive and interactive three-dimensional environment of the computer generated image was projected giving the visual impression of walking over a real bridge. Kinematic data were gathered with a motion capture system (MCS; Qualisys Oqus camera series) and used to update the computer generated image and to control the treadmill belt speed allowing for its automatic adjustment to the speed preferred by the walker. A fall arrest system was installed to prevent injury to the pedestrian. Integration of all these systems allowed the attainment of a safe, controllable, interactive and perceptually-representative experimental setup for examination of human-structure interaction on laterally oscillating structures.

For further details the reader is referred to Bocian [23].

### 2.1 Motion base-mounted instrumented treadmill

Avoidance of spatial restraints is especially important when investigating perturbed gait to permit walkers to adjust their gait and capture their natural behaviour. Therefore, a requirement of a generous walking area, chosen here as 2 m long by 1.5 m wide, governed the design dimensional criteria. A crucial structural design objective was low deflection tolerance, hence high stiffness, which was needed to ensure high natural frequencies of the treadmill in order to avoid polluting measured signals with errors due to resonance and flexure.

A 1.5 kW AC geared motor was chosen to drive the treadmill belt. Toothed belt and pulleys were used to prevent motor belt slippage and ensure ample responsiveness of the driving mechanism to the demands of the control system. The large diameter of the rollers benefited the design due to the “flywheel effect”, i.e. the large moment of inertia of the rotating mass helping to minimise variations in the angular velocity due to fluctuations in the drive and load over each step cycle. Moreover, the large contact area between the belt and the rollers helped to prevent excessive treadmill belt slippage. A motion capture system (Qualisys Oqus camera series) consisting of 6 infrared cameras and a data processing unit was utilised in the setup allowing kinematic data to be collected.

A custom motor speed controller was developed allowing the automatic adjustment of the speed of the belt to that of the subject, making provisions for the fore-aft position of the subject on the treadmill. To avoid adding complexity to the experimental setup, an output from the MCS was utilised as an input to the control algorithm. A simple speed control approach was chosen, based on a proportional relation between the motor acceleration and the speed variation of the subject estimated from the first derivative of the measured fore-aft position error from the target position on the treadmill, and realised with a proportional-derivative controller. After the appropriate adjustment of gains (proportional gain and derivative gain) the control system was found to fulfil all of its design objectives without inhibiting natural walking. This important issue is further discussed in Section 2.4, presenting performance assessment of the setup, and Section 3.2.3, after introducing the data collection and processing methods.

**Fig. 2:**
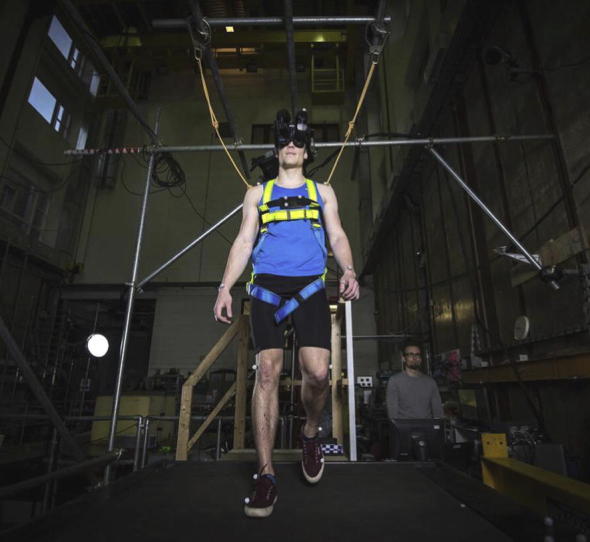
A subject during a test on the novel experimental setup developed at the University of Bristol.

The uniaxial shaking table utilised in the experiments is located in the Earthquake Engineering Laboratory at the University of Bristol, UK (see Fig. 2). Direct measurement of the GRFs is conducted using four Kistler 9327C piezoelectric force transducers. The drift in the lateral force signal associated with using piezoelectric technology, measured over 5 min, was 0.0011 N/s. Since each walking test conducted on the treadmill lasted approximately 3 min and the amplifier was reset prior to each test, the maximum error in the measured lateral force was below 0.2 N and is considered negligible. The force transducers provide a link between the treadmill and the small frame, and all components of the treadmill are otherwise mechanically isolated from other components of the setup.

### 2.2 Virtual reality

**Fig. 3:**
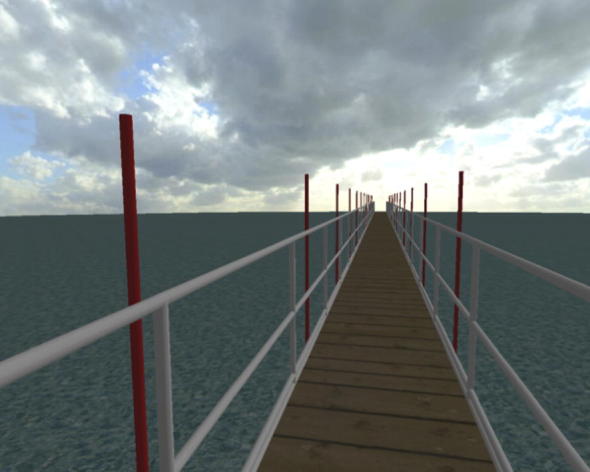
An example of the computer generated image projected onto the left-eye display of the head mounted display.

The developed virtual reality environment (VR; see Fig. 3) consists of three main elements; the bridge, the substratum (water/ground beneath the bridge) and the superstratum (sky). The 1.4 m wide bridge (0.1 m narrower than the treadmill belt) was positioned 4 m above the substratum. Its surface was rendered as a wooden deck to match the perceptual experience of walking on the medium-density fibreboard deck placed under the treadmill belt, thus enhancing the realism of the VR. The 1.2 m high handrails each side were built from 0.05 m diameter tubes with three horizontal rails and vertical upstands at 2 m centres. The substratum had posts positioned every 4 m symmetrically on both sides of the bridge to increase motion parallax. These posts, whose absolute position (i.e. relative to the ground) was stationary, might be thought of as lamp posts or any other stationary elements of the surrounding environment. The superstratum was a sunny sky with some clouds present, thus representing typical British conditions.

The computer generated image is projected in a stereoscopic HMD with 76° horizontal by 64° vertical field of vision and 1280 by 1024 resolution per eye (nVisor SX111). The headset has a mass of 1.3 kg and a total field of view of 102° horizontally and 64° vertically. This is smaller than the natural field of view of human binocular visual system which spans approximately 200° horizontally and 135° vertically [24]. However, it has been found that increasing the horizontal field of view does not substantially improve performance [25] and does not significantly affect step width and walking speed [26]. To account for other perceptual issues associated with use of the HMD, the device was chosen in compliance with recommendations by Patterson et al. [27]. For further discussion of these issues the reader is referred to Bocian [23].

### 2.3 Data acquisition

The data acquisition system used a 16 bit 64 channel Measurement Computing USB-2533 analogue-to-digital capture board, capable of sampling at 1MSample/s. The captured analogue data included the force-proportional signal from the force transducers, the velocity-proportional signal from the motor drive and the acceleration-proportional signal from a Sétra 114A capacitive uniaxial accelerometer attached to the side beam of the treadmill and aligned with the direction of the shaking table operation. Its signal was amplified by Fylde FE-254 low noise amplifier. An eighth-order Butterworth low-pass anti-aliasing filter (Kemo VBF 29) with cut-off frequency 40 Hz was applied to all analogue signals before data acquisition. The analogue data and MCS data were sampled at 128 Hz.

### 2.4 Performance assessment

A number of tests were conducted in order to understand the characteristics of the setup and validate its ability to measure lateral GRFs.

The variation in belt speed due to pedestrian loading during the tests with imposed belt speed (rather than belt speed feedback control, in order to allow comparison with the results by Savelberg et al. [28]) was below 3% (coefficient of variation), for a male subject with a mass of 81 kg and height 1.83 m walking at speeds ranging from 0.77 m/s to 1.75 m/s. This is considered acceptable [28].

An important issue in the performance assessment of the setup was validation of the treadmill belt speed feedback control mechanism. This is because, in order to ensure normal walking, the belt accelerations necessary to respond to variations in the test subject’s walking speed need to be minimised, such as not to cause undesirable perturbations to the walker. This issue will be discussed in Section 3.2.3, after introducing the data acquisition and processing methods. However, it should be noted here that the mean coefficients of variation of stride time and stride length in all tests reported in this study in which treadmill belt speed feedback control was engaged but the treadmill was unactuated (i.e. stationary in the lateral direction), were, respectively, 1.3% and 1.6%. These values are comparable to those reported by Jordan et al. [29] for subjects walking at their preferred (fixed) speed on a treadmill (cf. Fig. 3 in [29]).

The lowest lateral and vertical natural frequencies of the setup were identified at 13 Hz and 15 Hz, respectively, and were deemed far enough from the frequencies of interest below 6 Hz not to cause undesired pollution of the measured signals.

**Fig. 4:**
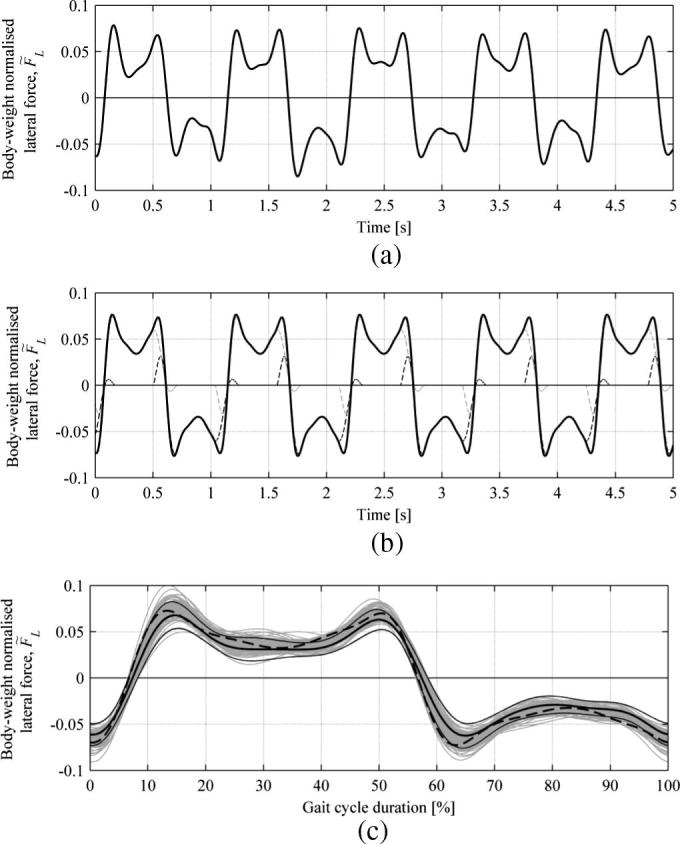
(a) Truncated time history of the measured lateral force from the treadmill. (b) Synthesised lateral force from force plate measurements. Black and grey dashed curves denote force traces from contralateral legs. (c) Comparison of the lateral force measured on the treadmill and force plate. Thick continuous curve denotes mean force measured over 95 gait cycles. Thin continuous curves denote values at two standard deviations from the mean. Dashed curve denotes reconstructed force obtained from a force plate. All data are for a male subject with $m_{p}=81\;\text{kg}$ and h=1.83m.

A dedicated experimental campaign was conducted to establish whether the lateral force measured on the treadmill was representative of real pedestrian behaviour, in a manner similar to that used by Carroll et al. [21]. For that purpose measurements were taken from a force plate (Kistler 9287CA equipped with a Kistler 5233A amplifier), which is considered the gold standard for obtaining GRFs. One male subject with a mass of 81 kg and height 1.83 m was asked to walk twenty times at his preferred speed, measured at approximately 1.55 m/s, along a walkway containing the force plate, located in the Bristol Vision Institute Motion Laboratory. A force trace from one leg was recorded during each test trail. A similar test was conducted, after a ten minutes habituation period, on the laterally stationary (unactuated) treadmill at an imposed speed corresponding to the speed during the tests on the force plate, with a difference in that the force measured was in this case continuous over multiple steps. This means that rather than measuring force traces from individual legs separately, combined force traces from both legs were recorded. To allow comparison between the results, the lateral force record from the treadmill was first filtered with a two-way fourth-order Butterworth low-pass filter with cut-off frequency 6 Hz and then, after rejecting transients associated with gait and treadmill initiation and termination stages, divided into gait cycles starting and ending at the heel strike of one of the legs. Since the timing of footsteps in normal gait is near but not perfectly periodic, the force records from individual gait cycles were time-normalised to fit within the common time scale chosen to represent the percentage of gait cycle duration. The temporal (and spectral) error introduced in the force profile due to this linear scaling process (cf. Racic and Brownjohn [30]) is negligible. For example, the resultant 95 body-weight normalised force records from a force signal recorded during a single trial on the treadmill are presented in Fig. 4(c) and denoted by grey curves. The mean gait cycle duration was 1.064s and the root-mean-square (RMS) error in gait cycle duration measured for these data was below 0.007s. Also presented in Fig. 4(c) is the mean force, denoted by a thick black curve, and values at two standard deviations away from the mean, denoted by thin black curves.

A representative record of lateral force during a gait cycle had to be generated from data measured from the force plate. For this purpose an average force trace for a step was established from these data. A continuous force record was then synthesised by overlapping this force trace by the average double stance duration identified in data from the treadmill, and it was then filtered in the same manner as for data from the treadmill. The resultant time history is presented in Fig. 4(b). A truncated force record of one gait cycle duration was extracted from the data in Fig. 4(b) and time-normalised as for the data in Fig. 4(a). This is represented in Fig. 4(c) by a dashed curve.

It can be seen in Fig. 4(c) that the synthesised force from the force plate generally falls between the curves indicating ± two standard deviations from the mean force measured on the treadmill and the characteristic “M” pattern is preserved. Based on this comparison, the ability of the instrumented treadmill to capture the GRFs of real pedestrian behaviour is considered satisfactory.

## 3 Experimental campaign

The experimental campaign, using human subjects, was granted approval by the University of Bristol Ethics of Research Committee.

### 3.1 Materials and methods

Basic data for six subjects who volunteered in the tests are given in Table 1. The cohort of subjects involved in this study represents a relatively young age group. This needs to be borne in mind in the assessment of the experimental results. Specifically, age-related changes in gait are associated with a decrease in preferred walking speed, stride frequency and length [31]. All subjects were naïve to the purpose of the experimental protocol and without neurological disorders and deficiencies in locomotor functions, and any other conditions which could impair their performance. All subjects were experienced with walking on a treadmill prior to participating in the study. To avoid errors associated with the subject’s predictive behaviour and any bias from changing behaviour with time, the tests were conducted in a random order. Each test started with the treadmill and the pedestrian stationary, enabling the output of the load cells for zero lateral force to be determined. Each test lasted for approximately three minutes which, after discarding transient periods due to gait and vibration initiation and termination stages, gave approximately two minutes of data for further processing. The subjects were instructed to walk at their comfortable speed and no other instructions were given.

Each subject wore their own gym-type shoes, which left their ankles uncovered, and tight fitting gym-type clothes, which allowed for placement of retro-reflective markers for the MCS close to their body. Each subject was fitted with a safety harness and then had their height and mass measured. Therefore, the measured mass included the subject mass, the mass of all clothes and shoes worn by the subject, the mass of markers and the harness. In the quantification of self-excited forces, the equivalent added damping and mass were normalised by the pedestrian mass inclusive of the mass of the HMD (1.3 kg) and proportion of the mass of a cable connecting the HMD to the image processing unit (1 kg). The belt speed feedback control allowing automatic adjustment of the treadmill speed to that of the walker was used in all experiments. Subjects were given at least ten minutes for habituation to walking on the treadmill in different combinations of vibratory and visual conditions imposed during the tests.

Considering the extent of the conducted tests outlined in the previous section, the following acronyms are used to denote visual and treadmill lateral motion conditions:

• VR (virtual reality) & NVR (no virtual reality, i.e. no HMD so subjects see the laboratory environment) for the cases in which the pedestrian walked with and without the HMD, respectively.
• NTLM (no treadmill lateral motion) & TLM (treadmill lateral motion) for the cases in which the pedestrian walked on stationary and laterally oscillating ground, respectively.

In all TLM tests sinusoidal treadmill motion was applied with amplitude A=0.01m and frequency $f_{b}=0.9\;\text{Hz}$. Only one set of vibratory conditions was used in this study, as explained in Section 1.3.

### 3.2 Data collection and processing

#### 3.2.1 Kinematic data

Pedestrian kinematic data were obtained with the MCS via proprietary software (Qualisys Track Manager) from seventeen markers placed on the subject’s body. Three of these markers were attached to a plate worn by the subject at the chest level and used to determine the subject’s fore-aft position on the treadmill for application with the treadmill belt speed feedback control. In addition, the MCS was used to capture data of the treadmill, belt and the HMD motion.

**Fig. 5:**
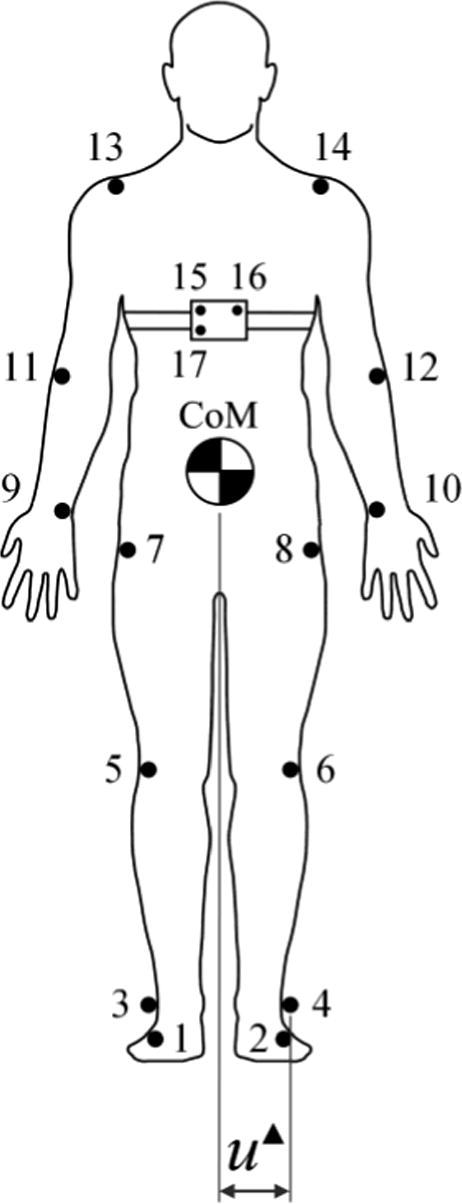
Locations of seventeen markers attached to the subject’s body, here shown in the frontal plane. Markers 3–14 were used to establish a nine-segment model for determination of the three-dimensional motion of the CoM and gait parameters. Markers 15–17 were used in treadmill belt speed controller. The centre of mass (CoM) and pseudo step width evaluated at the instant of heel strike ($u^{▴}$) are also denoted in the figure.

The CoM motion was obtained from kinematic data using a procedure outlined by Winter [32] (*linked-segment modelling*), by treating the human body as an assembly of rigid bodies (kinematic chain). It was assumed in the analysis that the mass of each segment and the location of its CoM relative to its joints remain fixed during movement. Nine body segments were defined from fourteen body markers for each subject placed at the fourth metatarsal bone (1 & 2), lateral malleolus (3 & 4), femoral condyles (5 & 6), greater trochanter (7 & 8), ulnar styloid (9 & 10), elbow axis (11 & 12) and glenohumeral axis (13 & 14), as can be seen in Fig. 5. In addition, Fig. 5 also shows the location of markers used in treadmill belt speed feedback control (15–17).

Each segment’s mass, location of its CoM relative to the proximal (nearer to body midline in kinematic chain made of body segments) segment end representing a joint were then identified using anthropometric data (cf. Table 4.1 in Winter [28]). The three-dimensional coordinates of the subject’s CoM were then calculated from the weighted average of the centres of mass of each segment.

#### 3.2.2 Identification of gait cycle events and determination of gait parameters

Heel strike events (also referred to as touchdowns) were detected in velocity signals obtained from the ankle marker (markers 3 & 4 in Fig. 5) vertical displacement signals following the method of O’Connor et al. [33]. The displacement signals were first filtered with a two-way fourth-order Butterworth low-pass filter with cut-off frequency 7 Hz, then numerically differentiated. The velocity signals obtained in such a way show a characteristic pattern repeatable for each gait cycle. An algorithm was written for automatic detection of one of the local minima within those patterns, which corresponds to the heel strike event identified in the measured lateral force signals for walking on stationary ground, filtered in the same manner [33].

Due to the unavailability of data about the location of the centre of pressure (CoP), the pseudo step width, $u^{▴}$, was taken as the medio-lateral distance between the position of the CoM, $x_{\text{CoM}}$, and the position of the lateral malleolus marker (hereafter referred to as an ankle marker; see Fig. 5, markers 3 and 4), $x_{\text{ANKLE}}$, at the instant of the heel strike:

(1) $$u^{▴}=x_{\text{ANKLE}}-x_{\text{CoM}}$$

The above relationship, depicted in Fig. 5, describes a pseudo step width as the CoP at the step initiation is known to be confined to the area underneath the heel of the foot [34] and step width was defined in the IPM as the distance between the lateral positions of the CoM and CoP at the instance of heel strike. However, due to anatomical constraints, this systematic simplification should allow the results to be representative of pedestrian stepping behaviour and compatible with the IPM.

Stride length was taken as the anterior–posterior distance between the locations of the ankle markers at the instances of two consecutive heel strikes for the same leg, accounting for the motion of the treadmill belt as measured by the MCS.

Stride duration was taken as the time between the instances of two consecutive heel strikes for the same leg. Stride frequency, also referred here as the lateral walking frequency, was taken as a reciprocal of the stride duration.

#### 3.2.3 Determination of a suitable length of signal for data processing

**Fig. 6:**
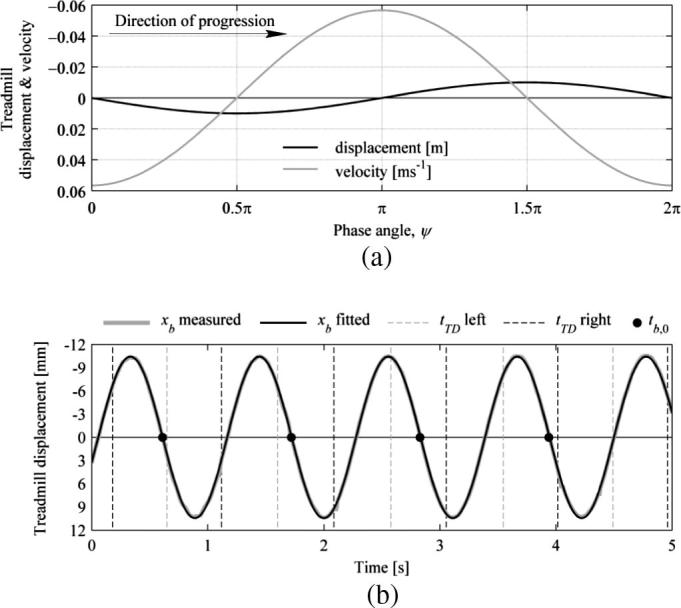
(a) Adopted convention for the phase angle ψ between the instance of the touchdown and the beginning of the nearest proceeding bridge vibration cycle defined here in relation to the bridge vibration period. (b) Truncated time histories of the measured and fitted treadmill lateral motion together with the identified instances of left and right leg touchdowns and beginnings of bridge vibration cycles for Subject 5 NVR TLM ($f_{b}=0.9\;\text{Hz}$, A=0.01m, $f_{p}=1.063\;\text{Hz}$).

For convenience and for consistency with previous modelling work [35], a phase angle between the bridge and pedestrian motion was used herein which was defined as:

(2) $$\psi =2\pi (t_{\mathrm{TD}}-t_{b\text{,}0})f_{b}$$

where $t_{\mathrm{TD}}$ is the timing of the leg touchdown and $t_{b\text{,}0}$ is the timing of the beginning of the nearest proceeding treadmill lateral vibration cycle occurring when the treadmill displacement is zero and velocity is at its positive maximum (positive defined as treadmill motion to the right of the walker; see Fig. 6(a)). Hence compatible phase angles for the right and left leg touchdowns are π apart. The phase angles for both legs in which those for the left leg are shifted by π and they are all then wrapped in order to keep them within the range $\langle 0\text{;}\;)\left(2\pi \right)$ are hereafter referred to as the compatible phase angles and are denoted by the symbol $\psi_{c}$.

In implementation of the algorithm, the treadmill lateral motion was taken from the MCS marker attached to the treadmill deck and a sine wave was fitted to the data using the least-square method. This was performed in order to establish precise timing of the beginnings of treadmill lateral vibration cycles. An example application of phase angle identification procedure is presented in Fig. 6(b) for data for Subject 5 NVR TLM ($f_{b}=0.9\;\text{Hz}$, A=0.01m, $f_{p}=1.063\;\text{Hz}$). It can be seen that the fitted treadmill lateral displacement data matches the measured data very well. The identified instances of left and right leg touchdowns are denoted in Fig. 6(b) by, respectively, grey and black dashed vertical lines and the beginnings of bridge vibration cycles are denoted by black dots. In the spectral calculations, in order to avoid leakage the signals were truncated such as to contain an integer number of pedestrian gait cycles. This was achieved by taking the longest possible interval within the record length between heel strikes on the same leg, after discarding transients associated with gait initiation and termination stages, which were assessed for each test from the recorded velocity of the treadmill belt. This was a sufficient condition for all NTLM cases, however, additional provisions had to be made for all TLM conditions. In those cases the length of the signal to be processed was chosen such that, in addition to containing an integer number of gait cycles, it also contained, as closely as possible, an integer number of bridge vibration cycles. This was achieved by utilising the phase angle data and finding the longest possible interval within the record length for which the difference between the initial phase angle at the heel strike and the final phase angle at the heel strike on the same leg was minimal, typically within 0.03π.

**Fig. 7:**
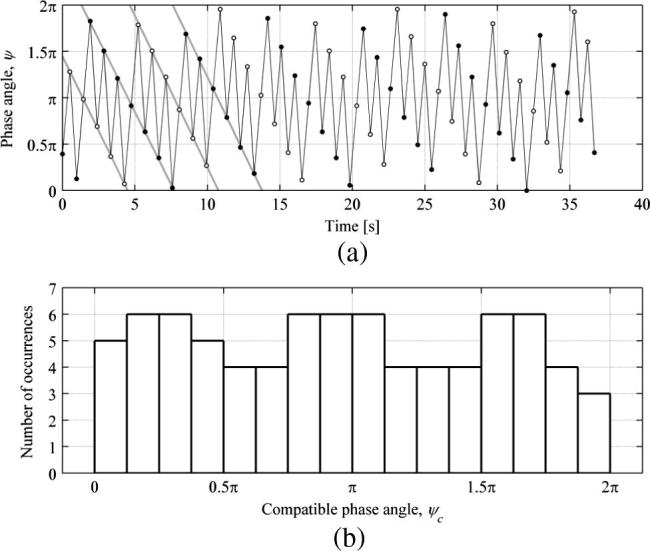
(a) Evolution of the phase angle between the instance of the right-leg touchdown and the beginning of the nearest preceding bridge vibration cycle, relative to the bridge cycle duration. Successive steps are joined by black lines. Best fit straight lines joining successive steps on the right (●) and left (○) foot are shown in grey. (b) Histogram of phase angles corresponding to the truncated data presented in (a). All presented data are for Subject 5 NVR TLM ($f_{b}=0.9\;\text{Hz}$, A=0.01m, $f_{p}=1.063\;\text{Hz}$).

**Fig. 8:**
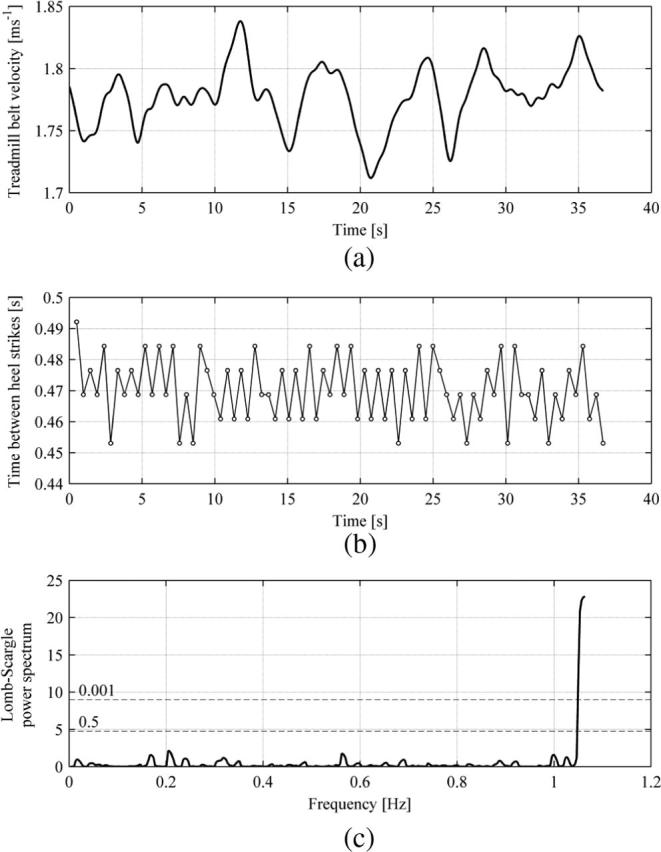
Variation over the test duration of the (a) treadmill belt velocity (low pass filtered at 1 Hz) and (b) time between consecutive heel strikes. (c) Lomn-Scargle power spectrum of time between heel strikes from data in (b). All presented data are for Subject 5 NVR TLM ($f_{b}=0.9\;\text{Hz}$, A=0.01m, $f_{p}=1.063\;\text{Hz}$).

In all the tests from which results are presented herein it was found that the pedestrian walking frequency was different from the treadmill lateral oscillation frequency and little affected by the treadmill motion. This is significant in that synchronisation of pedestrian walking frequency to the lateral oscillation frequency of a bridge is the most often purported mechanism causing exponential built-up of lateral vibration amplitudes. These results suggest that this might not be pronounced, at least for the vibratory conditions tested. Therefore, accounting for the phase drift (i.e. monotonic evolution of the phase angle according to the beating frequency: $\left|f_{p}-f_{b}\right|$), the data were truncated such as to cover all possible phase angles evenly. An example of the described procedure is shown in Fig. 7, based on data for Subject 5 NVR TLM. It can be seen in Fig. 7(a) that the phase angle for the right and left leg touchdowns, denoted by ● and ○, respectively, follows, to high accuracy, straight lines. Fig. 7(b) shows histograms of compatible phase angles, corresponding to the data in Fig. 7(a), with a bin size of π/8. No appreciable bias towards any range of phase angles exists in the presented data. Small differences between the counts of $\psi_{c}$ in different bins are associated with within-subject variability, which is presented in Fig. 8. Fig. 8(a) shows variation of the treadmill belt speed for Subject 5 NVR TLM, corresponding to data in Fig. 7, which was in response to the pedestrian behaviour through the feedback control system. Fig. 8(b) shows the corresponding variation in time between consecutive heel strikes with ordinate resolution of 1/128Hz=0.0078s. It is clear that there was slight variation in pedestrian walking frequency. Further analyses of time histories of the time between heel strikes were conducted by looking at the frequency content of these signals. Because the data are not evenly sampled (i.e. the time between consecutive heel strikes of the same leg is variable) FFT methods could not be used. Instead, the Lomb-Scargle algorithm [36] was applied to show the frequency components of the signals. The advantages of this method for analysing unevenly sampled data is that it does not require interpolation, which can often introduce spurious power at low frequencies, and it allows the significance of the identified components of the spectrum to be readily estimated [37]. Before obtaining the power spectrum, the algorithm requires normalisation of the data by subtracting the mean. Typical results of this evaluation are shown in Fig. 8(c). It can be seen that no component of the spectrum exceeds a significance level of 0.5, which marks an upper boundary for components that could be expected by chance. Therefore, the operation of the controller does not cause any statistically significant undesirable fluctuations in the pacing frequency of the walker.

It needs to be borne in mind that only one set of vibratory conditions was investigated in this study. It is plausible that synchronisation could occur at larger vibration amplitudes, which might affect the magnitudes of the forces exerted by pedestrians on structures. Further discussion of this issue is given in Section 4.3.

#### 3.2.4 Determination of self-excited forces

The self-excited forces at the bridge vibration frequency can be conveniently divided into two components, one in phase with bridge velocity and the other in quadrature to it, which, after appropriate scaling, can be included as equivalent added damping (ΔC) and mass (ΔM) (e.g. [4]) or stiffness (e.g. [14]) to the equation of motion of the structure. In order to find ΔM and ΔC from the pedestrian, for sinusoidal bridge motion, the average self-excited forces at the vibration frequency were determined by considering the transfer function between the measured acceleration and lateral force at that vibration frequency. For the vibratory condition used, two tests were conducted with the same motion but with the treadmill empty and the belt set to two different speeds. The component of the transfer function at the vibration frequency, ${\overset{⌢}{H}}_{{\overset{¨}{x}}_{b}F_{L}}(f_{b})$ was then identified from the measured data. Since the signals contained an integer number of vibration cycles the transfer function was simply found from the ratio of the FFTs of the force and acceleration, treated as one rectangular window thus avoiding spectral leakage. The same method was used to obtain the corresponding components of the transfer function for the pedestrian walking on the treadmill, $H_{{\overset{¨}{x}}_{b}F_{L}}(f_{b})$. The equivalent added mass, ΔM, and the equivalent added damping, ΔC, were then calculated from:

(3) $$\Delta M=-\text{Re}\lceil H_{{\overset{¨}{x}}_{b}F_{L}}(f_{b})-{\overset{⌢}{H}}_{{\overset{¨}{x}}_{b}F_{L}}(f_{b})\rceil$$

(4) $$\Delta C=\text{Im}\lceil H_{\overset{¨}{x}F_{L}}(f_{b})-{\overset{⌢}{H}}_{\overset{¨}{x}F_{L}}(f_{b})\rceil \omega_{b}$$

where Re⌈•⌉ denotes the real part and Im⌈•⌉ denotes the imaginary part of the complex number •, $\omega_{b}$ is the angular vibration frequency of the bridge and ${\overset{⌢}{H}}_{\overset{¨}{x}F_{L}}(f_{b})$ was taken from the empty treadmill test with the better match with the pedestrian walking velocity during the test. The presented values of ΔC and ΔM were normalised by pedestrian mass, $m_{p}$, giving: $\Delta \overset{∼}{C}=\Delta C/m_{p}$ and $\Delta \overset{∼}{M}=\Delta M/m_{p}$.

For other analysis of the measured forces a two-way fourth-order Butterworth low-pass filter with cut-off frequency 6 Hz was applied to all force data measured via force transducers. In order to obtain the time history of the pedestrian lateral force for the case of walking on moving ground, a time history of the lateral force for the empty treadmill vibrating at 0.9 Hz with amplitude of 0.01 m, with the belt running, was first determined and filtered as above. It was then subtracted from the measured time history of the total lateral force in the presence of the walker, after performing time-alignment.

## 4 Results and discussion

In this section the results of the experimental campaign are presented and related to the assumptions behind and predictions of the IPM analysed in [4], [9], [10]. Time histories of data on pedestrian behaviour while walking on stationary and laterally oscillating ground in NVR and VR conditions are first analysed in the context of pedestrian balance adjustments. The mechanism leading to the net input of energy into the vibrating mode is revealed based on empirical data. Components of pedestrian lateral force are identified. The effects of visual environment and ground condition on spatial and temporal gait parameters and self-excited forces are statistically tested.

Although the data presented in Sections 4.1, 4.2 relate to Subject 5, the relationships discovered in the data are applicable for all participants of the experimental campaign.

### 4.1 Pedestrian walking behaviour

**Fig. 9:**
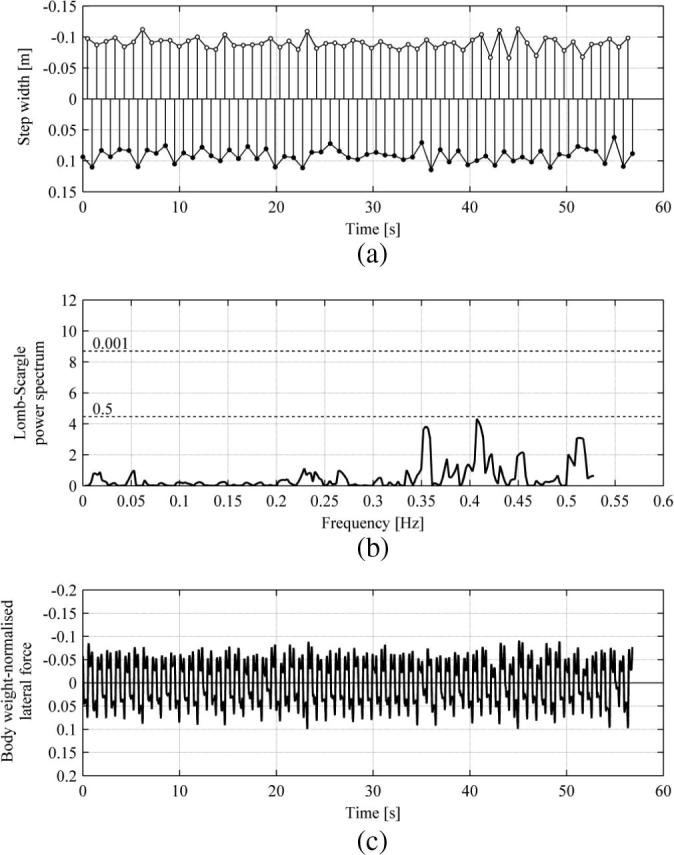
Results for Subject 5 walking in NVR NTLM. Data for the right and left legs are denoted by ● and ○, respectively. (a) Time history of the step width. (b) Lomb-Scargle power spectrum of the step width based on data from the left leg. Significance levels are denoted by dashed lines. (c) Time history of the body weight normalised pedestrian lateral force.

**Fig. 10:**
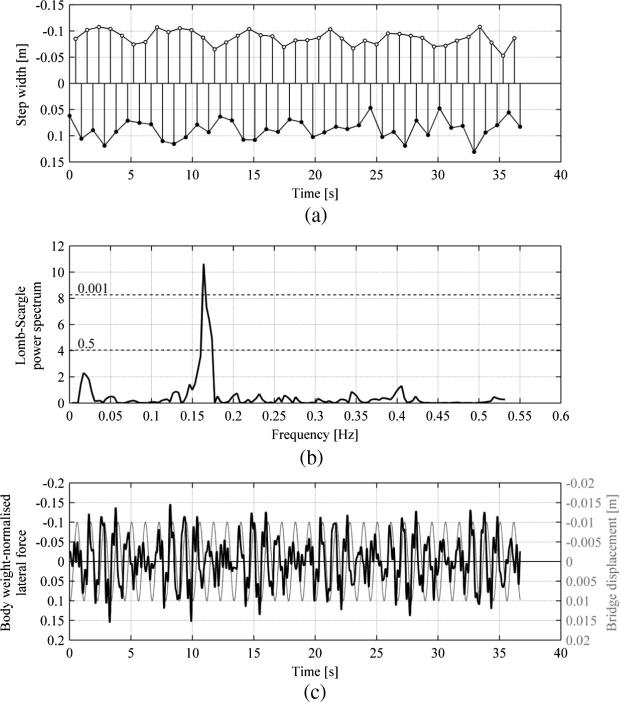
Results for Subject 5 walking in NVR TLM ($f_{b}=0.9\;\text{Hz}$, A=0.01m, $f_{p}=1.063\;\text{Hz}$). Data for the right and left legs are denoted by ● and ○, respectively. (a) Time history of the step width. (b) Lomb-Scargle power spectrum of the step width based on data from the left leg. Significance levels are denoted by dashed lines. (c) Time history of the body weight normalised pedestrian lateral force and treadmill displacement.

Typical time histories of the pedestrian behaviour while walking in NVR, in NTLM and TLM conditions are presented in Figs. 9 and 10, respectively, which were obtained for Subject 5. The different durations of the signals presented in the relevant plots in these figures are due to the requirement for the phase angles to be covered uniformly for TLM, as explained in Section 3.2.3. Figs. 9(a) and 10(a) show the evolution of the step width, $u^{▴}$, defined in Eq. (1), for the right (●) and left (○) leg. It can be seen that the values of $u^{▴}$ in Fig. 9(a) vary only little in comparison to the values in Fig. 10(a) and there is an underlying pattern of variation of data in Fig. 10(a) for each leg. In order to test the significance of variation of $u^{▴}$ frequency domain analysis was conducted on signals composed of data for one of the legs. Because the data are not evenly sampled (i.e. time between consecutive heel strikes of the same leg is variable), the Lomb-Scargle algorithm [36] was applied to show the frequency components of the signals (see Section 3.2.3). It can be seen in Fig. 9(b) that no component of the spectrum exceeds a significance level of 0.5 which marks the upper boundary for components which could be expected by chance. This implies that the variation of $u^{▴}$ in NVR NTLM has no significant pattern [37]. This is not the case for $u^{▴}$ in NVR TLM. It can be seen in Fig. 10(b) that there is a dominant sinusoidal component of the signal with a frequency of 0.163 Hz for which the significance level is greater than 0.999. This component, having a period of approximately 6.135 s, reveals itself in Fig. 10(a) in a wavelike pattern of the evolution of $u^{▴}$ for each leg. The signals corresponding to each leg contain six cycles of variation of $u^{▴}$ within their duration of approximately 37 s.

Time histories of body-weight normalised pedestrian lateral force are presented in Figs. 9(c) and 10(c) for NVR NTLM and NVR TLM, respectively. For the case of NVR NTLM, the amplitude of $F_{L}$ is quite constant throughout the record. For the case of NVR TLM, the ground motion obviously affects the motion of the CoM and as a result, since $f_{p}\neq f_{b}$, in line with the predictions of the IPM [4], $F_{L}$ is expected to be different from one step to another. This can be seen in Fig. 10(c) together with the bridge behaviour. $F_{L}$ indeed varies considerably for each step within the presented record both in amplitude and shape. The maximum force amplitude is approximately twice as large as the maximum force amplitude for NVR NTLM data in Fig. 9(c). Clearly, the pedestrian lateral force or, more generally, the pedestrian’s behaviour depends on their phase relative to the bridge motion.

**Fig. 11:**
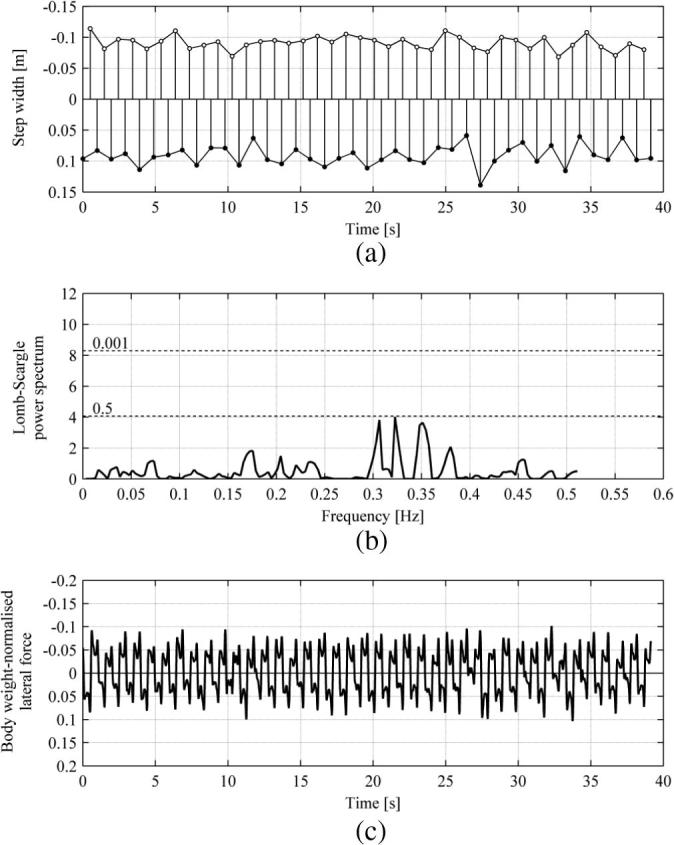
Results for Subject 5 walking in VR NTLM. Data for the right and left legs are denoted by ● and ○, respectively. (a) Time history of the step width. (b) Lomb-Scargle power spectrum of the step width based on data from the left leg. Significance levels are denoted by dashed lines. (c) Time history of the body weight normalised pedestrian lateral force.

**Fig. 12:**
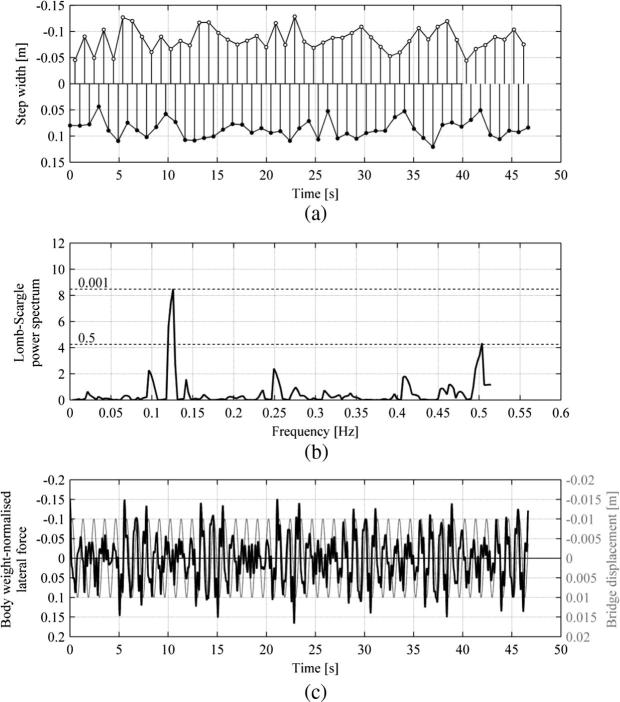
Results for Subject 5 walking in VR TLM ($f_{b}=0.9\;\text{Hz}$, A=0.01m, $f_{p}=1.029\;\text{Hz}$). Data for the right and left legs are denoted by ● and ○, respectively. (a) Time history of the step width. (b) Lomb-Scargle power spectrum of the step width based on data from the left leg. Significance levels are denoted by dashed lines. (c) Time history of the body weight normalised pedestrian lateral force and treadmill displacement.

The same analyses were conducted for data obtained from Subject 5 for walking in VR, in NTLM and TLM, for which the results are presented in Figs. 11 and 12, respectively. As in the case of walking on non-oscillating treadmill in NVR, no significant bias in step width data for VR, presented in Fig. 11(a), was discovered in the corresponding Lomb-Scargle power spectrum presented in Fig. 11(b). Furthermore, the pedestrian lateral force, presented in Fig. 11(c), was fairly consistent throughout the record, as is the case for data in Fig. 9(c).

Walking in VR TLM increased variability of step width in comparison to the corresponding NTLM test. This can be seen in Fig. 12(a). Fig. 12(b) presents Lomb-Scargle power spectrum of the step width data for one leg. A dominant sinusoidal component of the signal having a frequency of 0.129 Hz can be seen, for which the significance level reaches 0.999. This component, having a period of approximately 7.752 s, reveals itself in Fig. 12(a) in a wavelike pattern of evolution of $u^{▴}$ for each leg, however, it needs to be pointed out that this pattern is not as clearly visible as for the case of NVR TLM in Fig. 10(b). The signals corresponding to each leg contain six cycles of variation of $u^{▴}$ within their duration of approximately 47 s. The same pattern of variation is also apparent in Fig. 12(c) in body-weight normalised pedestrian lateral force.

The analysis of time histories of the gathered data revealed the modulating effect of the bridge motion on the pedestrian stepping behaviour and amplitude of pedestrian lateral force. Signal processing of the step width data using Lomb-Scargle power spectrum algorithm allowed patterns of variability of the step width to be rigorously identified. The dominant sinusoidal components of these patterns in data for Subject 5 walking in NVR and VR conditions with TLM occurred at 0.163 Hz and 0.129 Hz, respectively. The significance of these spectral components can be understood considering pedestrian stride frequencies, $f_{p}$, adopted in the tests. These were found at 1.063 Hz and 1.029 Hz for NVR TLM and VR TLM, respectively. The dominant components of the spectra are the beating frequencies, δf, between $f_{p}$ and $f_{b}$ (=0.9Hz). Therefore the step width (and lateral force) varies according to the phase between the pedestrian and bridge motion, which, for the case of phase drift, itself is a function of the beating frequency. This gives validity to the assumption behind the IPM adopted by the authors in their previous studies [4], [9], [10]. According to Hof et al.’s foot placement control law [6] the step width, which determines the magnitude of pedestrian lateral force is a function of the velocity of the CoM, which is affected by the bridge motion.

### 4.2 Components of the pedestrian lateral force

**Fig. 13:**
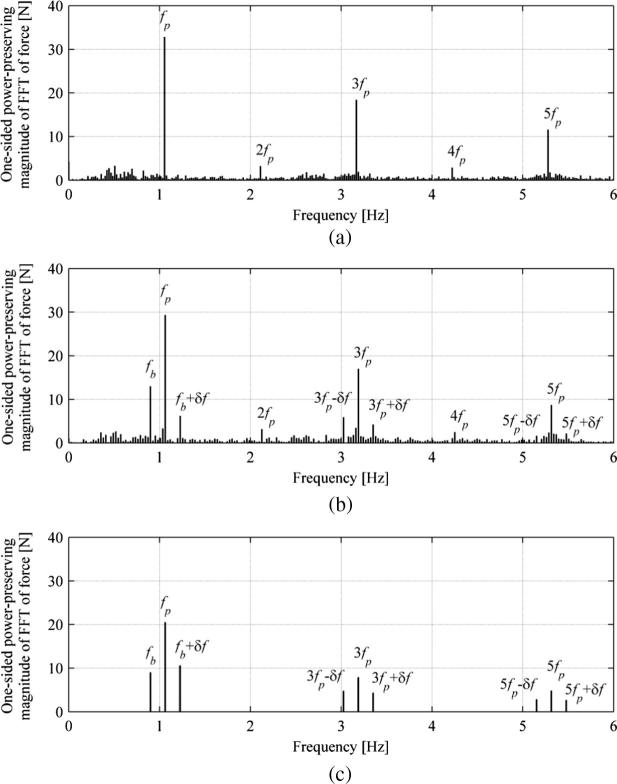
Frequency domain representation of the pedestrian lateral force signal for Subject 5 walking in (a) NVR NTLM ($f_{p}=1.056\;\text{Hz}$; frequency resolution: 0.021 Hz) and (b) NVR TLM ($f_{p}=1.063\;\text{Hz}$, $f_{b}=0.9\;\text{Hz}$, A=0.01m; frequency resolution: 0.027 Hz) conditions. (c) Output of the IPM, based on the absolute velocity foot placement control law, with the pedestrian parameters for Subject 5 ($m_{p}=67\;\text{kg}$, $l_{\text{eq}}=1.28\;\text{m}$ and $f_{p}=1.063\;\text{Hz}$) and $b_{\text{min}}=0.0157\;\text{m}$, and bridge parameters corresponding to data in (b). $\delta f=f_{p}-f_{b}$ is the beating frequency in (b) and (c).

Comparison of the results for Subject 5 NVR, showing typical behaviour observed during the tests, in terms of the lateral force is presented in Fig. 13. Fig. 13(a) and (b) contain one-sided power-preserving magnitudes of FFT of pedestrian lateral force for NTLM and TLM, respectively. The stride frequencies for NTLM NVR and TLM NVR were 1.056 Hz and 1.063 Hz, respectively, while the frequency resolutions of the plots in Fig. 13(a) and (b) are 0.021 Hz and 0.027 Hz, respectively. As expected, the components at odd harmonics of the stride frequency are visible in the measured data in Fig. 13(a) and (b). In addition, Fig. 13(a) and (b) also contain components at the first two even harmonics at $2f_{p}$ and $4f_{p}$ which indicates that the pedestrian force was not symmetric during the test, i.e. force traces from the right and left legs differed.

It can be seen in Fig. 13(b) that, in the case of walking on moving ground, the frequency components for walking on stationary ground are accompanied by additional components (i.e. side bands) located at $\delta f=f_{p}-f_{b}$ (i.e. the beating frequency) either side of them. They are particularly clear around the first and the third harmonic, although they can also be identified around the fifth harmonic.

The existence of side bands around the stride frequency and its harmonics is predicted by the IPM [4]. The corresponding output of the IPM for TLM NVR (A=0.01m and $f_{b}=0.9\;\text{Hz}$) for Subject 5 ($m_{p}=67\;\text{kg}$, $l_{\text{eq}}=1.28\;\text{m}$ and $f_{p}=1.063\;\text{Hz}$), with foot placement control law in which the CoM velocity is taken relative to a stationary point outside of the oscillating bridge and the constant offset is 0.0157 m, which is a typical value for walking on stationary ground, is presented in Fig. 13(c). The data were obtained from the generated lateral force time history corresponding to 127 bridge vibration cycles which ensured all phase angles were covered uniformly and there was also close to an integer number of pedestrian cycles (150) in the duration of the record. Hence no force components apart from the ones at the stride frequency and its odd harmonics, and self-excited forces, are visible in Fig. 13(c). It can be seen that predictions of the IPM qualitatively agree remarkably well with the data from measurements in Fig. 13(b). The only difference is the existence of force components at even harmonics of stride frequency in Fig. 13(b), which are associated with gait asymmetry.

Decomposition of pedestrian lateral forces obtained from experiments on laterally-oscillating ground was previously conducted by Pizzimenti and Ricciardelli [14], Ingólfsson et al. [12], [38], Ingólfsson [39] and Carroll et al. [21]. However, the self-excited forces were identified therein at best around the component at the stride frequency only. Moreover, a considerable spread of energy from the spectral components of force at the stride frequency and its odd harmonics, and those few identified self-excited force components into their neighbouring spectral bins was always evident, often claimed to be associated with the variability in pedestrian behaviour. It has to be pointed out here that in all of the abovementioned studies the velocity of the treadmill belt remained fixed during the test thus providing a strong stimulus for gait rhythmicity. It could be expected that the observed variability in pedestrian behaviour should have been lower than in the current study in which the pedestrian has been allowed to adjust their gait freely. Conversely, the new results presented in this section show that the effect of variability is not as strong as previously suggested. It is believed that the apparent spread of the spectral components in the previous studies was likely due to leakage. On the other hand the signal processing method adopted here has minimised leakage and yielded much cleaner plots of the frequency content of the forces in the presence of laterally oscillating ground.

Despite the remarkably good performance of the IPM qualitatively, based on the chosen parameters, it does not accurately predict the magnitudes of pedestrian force components. Therefore further work is necessary to reconcile this discrepancy.

The self-excited force component at the bridge vibration frequency is the most important since it can drive structural instability. Therefore, this component will be considered in more detail in the next section in which comparison between results from tests conducted in different visual and ground conditions is made.

### 4.3 Effects of visual environment and ground motion

The results of the tests for all subjects are presented in Table 2. The self-excited forces are given in terms of the equivalent added damping and mass normalised by pedestrian mass ($\Delta \overset{∼}{C}$ and $\Delta \overset{∼}{M}$, respectively), and gait parameters are given in terms of the stride time (i.e. reciprocal of stride frequency), stride length and (pseudo) step width, obtained with the procedures outlined in Section 3. The means and standard deviations of all the parameters are denoted in, respectively, normal and italic font style.

It can be seen from Table 2 that for Subjects 1–4 $\Delta \overset{∼}{C}$ was lower (hence more detrimental for structural stability) for VR than for NVR, but the opposite relation occurred for Subjects 5 & 6. The mean (over all subjects) was 16% lower. $\Delta \overset{∼}{M}$ was higher for VR than for NVR for all subjects, the mean (over all subjects) being 73% higher. The measured positive mean equivalent added mass per pedestrian is consistent with the observed decrease in vibration frequency identified by Brownjohn et al. [7] during a test on the CMB which experienced instability in a lateral structural mode at 0.9 Hz corresponding to the treadmill vibration frequency applied during the treadmill tests. This is different from the results from treadmill tests by Ingólfsson et al. [13] who suggested that this value should be close to zero at this frequency, in which case no shift in the vibration frequency would be expected. However, unlike in that study, in all the tests reported herein, the pedestrian stride frequency was different from the treadmill lateral vibration frequency and fairly constant throughout each test (see standard deviations of stride time in Table 2). It is plausible that some modulation of pedestrian stepping behaviour (e.g. phase pulling, phase locking or possibly synchronisation) could occur while walking on oscillating ground, conceivably due to a phase entrainment mechanism similar to the one proposed by Bocian et al. [35], for the case of vertical bridge vibrations, or by McRobie et al. [12]. Such a mechanism could bias the results towards those obtained by Ingólfsson et al. [13], but it is equally possible that this could make them differ even more. Unfortunately, at this point, there are no reliable data available to assess this issue. It needs to be pointed out that, considering all the TLM tests, the coefficients of variation of $\Delta \overset{∼}{C}$ and $\Delta \overset{∼}{M}$ were, respectively, 0.258 and 0.4375. These are much lower values than those obtained by Ingólfsson et al. [12], for the same lateral vibration velocity and acceleration amplitudes (cf. Fig. 13(a) and (b) for ${\overset{˙}{x}}_{b}=0.0566\;\text{m}/\text{s}$ and ${\overset{¨}{x}}_{b}=0.3198\;\text{m}/{\text{s}}^{2}$, respectively, in [13]), which were close to 3 and 10 for $\Delta \overset{∼}{C}$ and $\Delta \overset{∼}{M}$, respectively. The lower variability in the quantified self-excited forces in the current study is primarily believed to be due to the signal processing issues raised in Point 3 and outlined in Section 3.2.3, which were generally neglected in previous empirical studies in the field of human-structure interaction on vibrating ground utilising treadmills.

Introducing ground motion or the virtual reality environment, while keeping the other unchanged, usually had an effect of decreasing stride time (hence increasing stride frequency; 21 out of 24 pairs of tests) and increasing step width (17 out of 24 pairs of tests) by, on average, 1.3% and 2.2%, respectively. Higher variability of step width was caused by introducing ground motion in NVR and VR for all but one pairs of tests. This reflects the stepping behaviour and its modification by the bridge motion analysed in Section 4.1.

The mean walking velocity during each test can be obtained from the data in Table 3, by dividing the stride length by the stride time. Rather surprisingly, introducing ground motion had an effect of increasing the mean velocity, for both NVR (for 4 out of 6 subjects) and VR (for 5 out of 6 subjects), although the changes were small. Considering all pairs of tests for all subjects grouped with respect to the ground condition, the mean velocity for TLM was on average 3.25% higher than for NTLM with a maximum increase of 7.11%. This gives support to the importance of making allowance for the adaptability of human gait in laboratory investigations advocated in Point 2 (Section 1.2).

In order to test the statistical significance of these and other effects one-tailed dependent t-tests for paired samples were applied to the data in Table 2. The results of statistical analysis of the effects of ground and visual conditions on gait parameters and, where applicable, self-excited forces are presented in Table 3. The type of the conducted analysis is indicated in the first column, in which level 1 and level 2 manipulated independent variables (MIV; i.e. ground or visual conditions) are defined. For example, the first set of results in Table 3 is representative of tests during which the ground was stationary (NTLM) and visual conditions were varied (NVR & VR). This set of results will be hereafter denoted as NTLM (NVR & VR) and similar notations will be used for other sets of results in Table 3. A significance level of 5% (p=0.05) was adopted, meaning that the null hypothesis, stating that the means of both samples are the same (i.e. level 2 MIV have no influence on the examined variables), is rejected if their probability of being the same is less than 5%. The values of the *t*-statistic are also given in Table 3 for completeness. The sign of these values indicates the direction of the difference between sample means, i.e. positive and negative values of *t*-statistic indicate that the sample mean, respectively, decreased or increased from the first to the second condition assigned to level 2 MIV.

No statistically significant difference was found for any of the quantified gait parameters in the case of NTLM (NVR & VR) and NVR (NTLM & TLM). However, for NTLM (NVR & VR), stride time decreased from NVR to VR for 5 out of 6 subjects and the corresponding *p*-value was close to being significant at the adopted significance level of 5%.

In the case of TLM (NVR & VR) statistically significant difference was found for $\Delta \overset{∼}{M}$, which increased from NVR to VR. No statistically significant difference was found for any other parameter, although $\Delta \overset{∼}{C}$ and stride time were close to being significant at the adopted significance level of 5%.

In the case of VR (NTLM & TLM) statistically significant difference was found for all gait parameters except step width. Stride time and mean walking velocity increased, and stride length decreased from NTLM to TLM.

Considering all the information contained in this section, an important conclusion is that the capability of the pedestrian to freely adjust their gait should be provided in future studies in order to capture natural walking behaviour, as indicated in Point 2. Providing a visual environment offering information compatible with overground walking can have a significant effect on gait parameters and magnitudes of self-excited forces, as indicated in Point 1.

Further tests for a wider range of treadmill vibration frequencies and amplitudes would give a fuller picture of the human-structure interaction on laterally oscillating ground.

## 5 Conclusions

A novel experimental setup for the investigation of pedestrian actions on laterally oscillating ground has been presented which is currently state-of-the-art. An immersive and interactive virtual reality environment and motor speed feedback control mechanism have been included in the setup in order to ensure congruence of sensory information and facilitate natural walking behaviour. Analyses of the experimental data gathered from the setup have allowed the adaptation of step width to be confirmed as the strategy for controlling stability while walking on laterally oscillating ground. Importantly, this result comes from walking tests unbiased by imposed treadmill speed, as was the case in previous similar tests reported by others [21], [22]. The adjustment of step width has been assumed to be the main strategy controlling stability of the inverted pendulum pedestrian model in the presence of lateral ground vibration, and the results of the current study give evidence supporting this assumption.

Signal processing issues, neglected in past investigations, have been considered in the determination of the forces exerted by pedestrians on vibrating ground. Specifically, the requirement for phase angles to be covered uniformly in quantifying the average magnitudes of self-excited forces in the case of pedestrian stride and bridge vibration frequencies being different and constant throughout the test was taken into account. This is believed to be a predominant cause for lesser variability of self-excited forces obtained in this study, in comparison to previously reported results. Rigorous signal processing also enabled it to be shown for the first time in experimental data that the inverted pendulum pedestrian model qualitatively captures the nature of the pedestrian loading remarkably well, being capable of reproducing all components of the loading, including the self-excited forces located around the third and fifth multiple of the stride frequency. Improvements to the model, based on further experimental investigations, will facilitate more reliable modelling of pedestrian behaviour and refinement of dynamic stability criteria for bridges under pedestrian lateral action.

The influence of the ability to adjust gait freely and that of the quality of the available visual information on pedestrian behaviour has been examined. It has been found that introducing lateral ground motion caused an increase in walking velocity, regardless of whether the pedestrian was presented with the visual environment of the laboratory or the virtual bridge, thus justifying inclusion of the speed feedback control mechanism in the experimental setup. No statistically significant difference has been found for any parameters due to varying visual conditions while walking on stationary ground. Walking on vibrating ground in the visual conditions representative of walking on a real bridge caused a statistically significant change in the self-excited forces in comparison to walking in the visual environment of the laboratory. More importantly, it has been found that the magnitude of this effect can be significant in the context of structural stability. Statistically significant differences have been found in the gait cycle duration, stride length and walking velocity by introducing ground motion while walking in the visual environment of the bridge. This indicates the importance of providing visual information compatible with real life experience.

A new line of inquiry into the causes of instability of structures due to human actions has been opened in this study thanks to fusion of biomechanics and cognitive psychology. The advances owed to this approach render further utilisation of virtual reality technology warranted in studies on human behaviour in the built environment, in the context of vibration serviceability of structures.

## Figures and Tables

**Table 1:** Basic data for all experimental subjects.

ID	Gender	Age (years)	Height h (m)	Mass $m_{p}$ (kg)
1	Male	26	1.81	81
2	Female	20	1.64	51
3	Male	19	1.81	75
4	Male	21	1.93	81
5	Male	21	1.80	67
6	Female	20	1.82	68

**Table 2:** Results of the treadmill tests. Means and standard deviations of parameters are denoted in, respectively, normal and italic font style.

		Subject ID						Entire sample
		1	2	3	4	5	6	
*NVR*								
NTLM	Stride time (s)	0.9977	1.0209	0.8998	1.0526	0.9465	1.0492	0.9945
		*0.0143*	*0.0179*	*0.0100*	*0.0123*	*0.0095*	*0.0150*	*0.0553*
	Stride length (m)	1.6185	1.4516	1.6941	1.7495	1.6298	1.6354	1.6298
		*0.0254*	*0.0279*	*0.0145*	*0.0123*	*0.0261*	*0.0336*	*0.0916*
	Step width (m)	0.1198	0.0870	0.0879	0.1126	0.0870	0.1015	0.0993
		*0.0109*	*0.0080*	*0.0103*	*0.0092*	*0.0115*	*0.0104*	*0.1417*
TLM	Stride time (s)	1.0315	0.9923	0.8893	1.0378	0.9408	1.0460	0.9896
		*0.0748*	*0.0183*	*0.0088*	*0.0105*	*0.0103*	*0.0143*	*0.0573*
	Stride length (m)	1.5544	1.5078	1.6590	1.8044	1.6419	1.6352	1.6338
		*0.0991*	*0.0283*	*0.0221*	*0.0223*	*0.0392*	*0.0308*	*0.0931*
	Step width (m)	0.1296	0.0897	0.0900	0.1111	0.0862	0.1022	0.1014
		*0.0155*	*0.0113*	*0.0113*	*0.0122*	*0.0165*	*0.0123*	*0.0153*
	Normalised equivalentadded damping $\Delta C/m_{p}$ (s^−1^)	−1.6296	−1.7647	−2.1867	−1.0000	−2.6269	−1.5147	−1.7871
								*0.5138*
	Normalised equivalentadded mass $\Delta M/m_{p}$ (–)	−0.0494	0.2745	0.1600	0.3704	0.3881	0.2206	0.2274
								*0.1470*

*VR*								
NTLM	Stride time (s)	0.9764	0.9749	0.8893	1.0280	0.9783	1.0092	0.9760
		*0.0190*	*0.0160*	*0.0095*	*0.0097*	*0.0105*	*0.0136*	*0.0435*
	Stride length (m)	1.5163	1.5080	1.6855	1.8278	1.5271	1.7279	1.6321
		*0.0224*	*0.0326*	*0.0249*	*0.0239*	*0.0268*	*0.0428*	*0.1226*
	Step width (m)	0.1226	0.0893	0.0921	0.1131	0.0871	0.0976	0.1003
		*0.0139*	*0.0158*	*0.0383*	*0.0153*	*0.0150*	*0.0148*	*0.0131*
TLM	Stride time [s]	0.9636	0.9507	0.8888	1.0254	0.9721	1.0023	0.9672
		*0.0133*	*0.0130*	*0.0092*	*0.0101*	*0.0095*	*0.0093*	*0.0430*
	Stride length (m)	1.5416	1.5676	1.6827	1.8647	1.5622	1.7652	1.6640
		*0.0314*	*0.0220*	*0.0220*	*0.0301*	*0.0398*	*0.0295*	*0.1194*
	Step width (m)	0.1254	0.0938	0.1009	0.1128	0.0819	0.1063	0.1035
		*0.0174*	*0.0173*	*0.0393*	*0.0153*	*0.0201*	*0.0159*	*0.0138*
	Normalised equivalent added damping $\Delta C/m_{p}$ (s^−1^)	−1.6543	−2.1569	−2.3733	−1.4691	−2.5522	−1.4265	−1.9387
								*0.4428*
	Normalised equivalent added mass $\Delta M/m_{p}$ (–)	0.1852	0.4314	0.2800	0.3951	0.4030	0.3382	0.3388
								*0.0846*

**Table 3:** Results of statistical analysis of the effects of ground and visual conditions on the self-excited forces and gait parameters.

Level 1 MIV	Level 2 MIV	Computed terms	Normalised equivalent added damping $\Delta C/m_{p}$ (s^−1^)	Normalised equivalent added mass $\Delta M/m_{p}$ (–)	Stride time (s)	Stride length (m)	Step width (m)	Walking velocity (m/s)
NTLM	NVR & VR	*t*-statistic	n/a	n/a	1.6256	−0.0634	−0.8634	−0.5764
		*p*-value	n/a	n/a	0.0825	0.4759	0.2137	0.2946
TLM	NVR & VR	*t*-statistic	1.5563	−3.3013	1.5435	−1.0322	−0.8803	−1.3897
		*p*-value	0.0902	0.0107	0.0917	0.1747	0.2095	0.1117
NVR	NTLM & TLM	*t*-statistic	n/a	n/a	0.5654	−0.2020	−1.2615	−0.3745
		*p*-value	n/a	n/a	0.2981	0.4239	0.1314	0.3617
VR	NTLM & TLM	*t*-statistic	n/a	n/a	2.5219	−3.8317	−1.4568	−3.4800
		*p*-value	n/a	n/a	0.0265	0.0061	0.1025	0.0088
